# Ankle resistance with a unilateral soft exosuit increases plantarflexor effort during pushoff in unimpaired individuals

**DOI:** 10.1186/s12984-021-00966-5

**Published:** 2021-12-27

**Authors:** Krithika Swaminathan, Sungwoo Park, Fouzia Raza, Franchino Porciuncula, Sangjun Lee, Richard W. Nuckols, Louis N. Awad, Conor J. Walsh

**Affiliations:** 1 John A. Paulson School of Engineering and Applied Sciences, Harvard University, Boston, MA 02134 USA; 2 Sargent College of Health and Rehabilitation Science, Boston University, Boston, MA 02215 USA

**Keywords:** Resistance training, Gait biomechanics, Soft exosuit, Locomotor adaptation

## Abstract

**Background:**

Ankle-targeting resistance training for improving plantarflexion function during walking increases rehabilitation intensity, an important factor for motor recovery after stroke. However, understanding of the effects of resisting plantarflexion during stance on joint kinetics and muscle activity—key outcomes in evaluating its potential value in rehabilitation—remains limited. This initial study uses a unilateral exosuit that resists plantarflexion during mid-late stance in unimpaired individuals to test the hypotheses that when plantarflexion is resisted, individuals would (1) increase plantarflexor ankle torque and muscle activity locally at the resisted ipsilateral ankle, but (2) at higher forces, exhibit a generalized response that also uses the unresisted joints and limb. Further, we expected (3) short-term retention into gait immediately after removal of resistance.

**Methods:**

Ten healthy young adults walked at 1.25 m s^−1^ for four 10-min discrete bouts, each comprising baseline, exposure to active exosuit-applied resistance, and post-active sections. In each bout, a different force magnitude was applied based on individual baseline ankle torques. The peak resistance torque applied by the exosuit was 0.13 ± 0.01, 0.19 ± 0.01, 0.26 ± 0.02, and 0.32 ± 0.02 N m kg^−1^, in the LOW, MED, HIGH, and MAX bouts, respectively.

**Results:**

(1) Across all bouts, participants increased peak ipsilateral biological ankle torque by 0.13–0.25 N m kg^−1^ (p < 0.001) during exosuit-applied resistance compared to corresponding baselines. Additionally, ipsilateral soleus activity during stance increased by 5.4–11.3% (p < 0.05) in all but the LOW bout. (2) In the HIGH and MAX bouts, vertical ground reaction force decreased on the ipsilateral limb while increasing on the contralateral limb (p < 0.01). Secondary analysis found that the force magnitude that maximized increases in biological ankle torque without significant changes in limb loading varied by subject. (3) Finally, peak ipsilateral plantarflexion angle increased significantly during post-exposure in the intermediate HIGH resistance bout (p < 0.05), which corresponded to the greatest average increase in soleus activity (p > 0.10).

**Conclusions:**

Targeted resistance of ankle plantarflexion during stance by an exosuit consistently increased local ipsilateral plantarflexor effort during active resistance, but force magnitude will be an important parameter to tune for minimizing the involvement of the unresisted joints and limb during training.

**Supplementary Information:**

The online version contains supplementary material available at 10.1186/s12984-021-00966-5.

## Background

Independent and efficient locomotion has been linked with improved community participation and quality of life [1]. However, neuromotor disorders, such as stroke, a leading cause of disability, can disrupt the fine-tuned mechanics of walking [2]. More than 80% of people who have experienced a stroke are left with locomotor dysfunction, resulting in slow, asymmetric and unstable gait [3]. Weakened plantarflexor and dorsiflexor muscles in the impaired, or paretic, ankle are major contributors to these observed gait characteristics [4]. In particular, reduced muscle activity and ankle torque, collectively termed plantarflexor effort in this paper, during the stance phase has been linked to reduced propulsion [5], a key driver of walking speed [6]. As a result, many current rehabilitation programs focus on increasing paretic plantarflexor effort towards the high-level outcome of increasing gait speed through both conventional and robotic interventions. However, people poststroke can also attain higher gait speeds through compensatory mechanisms that compromise overall gait quality, such as hip hiking and circumduction, which rely on engaging proximal joints and the less-impaired, contralateral limb during the swing phase, rather than on increasing paretic ankle torque production during the stance phase [7, 8]. Thus, one aspect of a successful rehabilitation intervention is targeting increasing paretic plantarflexor muscle activity and biological ankle torque specifically during the pushoff phase of gait, towards achieving the functional outcome of increasing gait speed.

Resistance training during task-specific gait rehabilitation for people poststroke, such as walking with weights, is well-recognized as a method for increasing muscle strength [9] and paretic propulsion [10]. Resistance training further increases intensity during rehabilitation, which has been identified as a key contributor to improved rehabilitation outcomes alongside task-specificity and amount, where intensity is defined as the amount of mechanical work per unit time [11]. Physical therapy often incorporates resistance through techniques such as adding weights to the patient’s limb segments as they walk [12], practicing walking in water tanks [13], or pulling back on the patient as they walk with a resistive band attached to the pelvis [10]. However, such global methods load the entire limb rather than targeting the paretic joint and may lead to unintended responses such as increased torso or proximal joint involvement [10, 14]. Moreover, by applying a force throughout the entirety of the gait cycle, these methods do not specifically target the mid-late stance phase of gait during which most of the positive power required to propel the body forward is generated [15]. Thus, individuals may alter their gait patterns across the entire stride [16–19], thereby reducing the task-specificity of training. Recent studies have shown that by applying targeted resistance during only the stance or swing phase rather than throughout the stride, people poststroke further improved step length symmetry [20] or walking speed [21], respectively. However, this work has yet to be to be investigated with an approach that specifically targets the ankle during the stance phase towards increasing plantarflexor effort. Here, we aim to develop and validate such an approach in healthy individuals to examine feasibility and characterize the unimpaired response to a stance-specific and ankle-targeting resistive paradigm.

A growing number of wearable, assistive, joint-specific systems tailor applied force profiles to match biologically-relevant timings to target a specific joint *and* time within the gait cycle [22–25]. More recent work has started to investigate resistance training that is similarly both joint and phase-specific using passive [26] and active [27] approaches. Rigid devices such as those used in the aforementioned studies, however, present the added challenges of increased distal inertia and misalignment with the user’s joints, which effectively add load across the limb. In contrast, soft cable-driven exosuits developed by various groups have shown the ability to apply joint-specific torques while adding little mass or restrictions to the user’s limbs [25, 28, 29]. These devices are transparent when in the “slack” mode, during which the cables are not under tension, and induce minimal changes in gait from wearing the device alone [30, 31]. A recent study showed that using a soft unilateral exosuit to apply constant, low-force resistance at the ankle from late stance to mid-swing in healthy individuals led to changes in joint range of motion [32]. Another study found that walking overground with a passive, compliant, ankle resistive device resulted in adaptation of ankle joint velocity during the period of resistance, and posited that these changes may have implications for training increased plantarflexor muscle activity [33]. While these recent advances are moving towards joint and phase-specific paradigms, the influence of such targeted resistive approaches on the biomechanical drivers of gait kinematics, i.e., kinetics and muscle activity, has yet to be investigated in both healthy and clinical populations.

Conventional resistance training methods further typically vary task intensity through progressive schemes, such as increasing the band stiffness or adding higher weights to the foot over time [9, 12]. In contrast, most existing literature investigating joint and stance specific resistance with wearable devices do not systematically vary the applied resistance force magnitude [27, 32–36], and thus there is little knowledge of how resistance magnitude should be set to modulate intensity at the ankle. People poststroke and healthy individuals often rely on proximal joints or the contralateral limb to compensate for ankle weakness [37] or for high intensity tasks such as incline walking [20, 38]. Consequently, joint-specific resistive forces that are too high may result in overreliance on the unresisted joints, which negates the intent of targeting a specific joint. Hence, it is critical to identify the resistance level that results in minimal changes at the unresisted joints while increasing plantarflexor effort at the resisted ankle joint.

Our goal was to investigate the effect of stance-phase plantarflexor resistance on the kinetics of the resisted ankle joint, and the unresisted proximal joints and contralateral limb, across varying resistance magnitudes. Towards the eventual goal of developing strategies for poststroke gait rehabilitation, we investigated the response to targeted ankle resistance in healthy young subjects with a unilateral soft ankle exosuit previously developed by our group for poststroke gait assistance [39]. We elected to use a unilateral exosuit to enable characterization of interlimb tradeoffs in user response, an important consideration for future clinical applications. The unique ability of the soft exosuit to be instantaneously transparent when no forces are applied [40] further allowed us to obtain the effects of resistance on gait immediately after resistance is removed, without halting walking, to examine carryover effects. Such analysis of carryover effects have previously been used to indicate potential for stroke rehabilitation when resisting the pelvis [20] or imposing asymmetric walking constraints [41], and to provide evidence of feed-forward adaptation in healthy individuals [42].

In this study, we investigated the hypothesis that using an exosuit to resist plantarflexion during the stance phase would induce increased plantarflexor effort as measured by peak biological ankle torque and soleus activity at the resisted joint. We further posited that as resistive force magnitude increased, changes in kinetics at the unresisted proximal joints and contralateral limb would become apparent, reflecting the intralimb and interlimb tradeoffs towards generating the required biological torque. Finally, we expected carryover of observed changes in muscle activity and kinetics during active resistance into post-active gait, immediately upon removal of the applied resistance, given previous evidence for carryover after unilateral swing-phase resistance [43]. We then conducted two exploratory pilots to further understand the specificity of the resistive exosuit paradigm for increasing plantarflexor effort. We examined differences in the within-subject response when (1) using a resistive exosuit approach versus a conventional elastic band approach, and (2) providing explicit instructions to increase ankle plantarflexor effort.

## Methods

### Participants

Ten healthy young adults (n = 10; 3F, 7M; age = 28.5 ± 3.7 years (mean ± std); mass = 67.3 ± 11.2 kg; height = 1.72 ± 0.08 m) were recruited to participate in this study. Nine participants were right leg dominant [44, 45]. All participants were naïve to resistive exosuits. All participants reported no previous history of musculoskeletal injury or disease, and provided written informed consent prior to participation. The study was approved by the Harvard Longwood Medical Area Institutional Review Board, and all methods were carried out in accordance with the approved study protocol.

### Exosuit hardware and control

#### Apparel

**Fig. 1:**
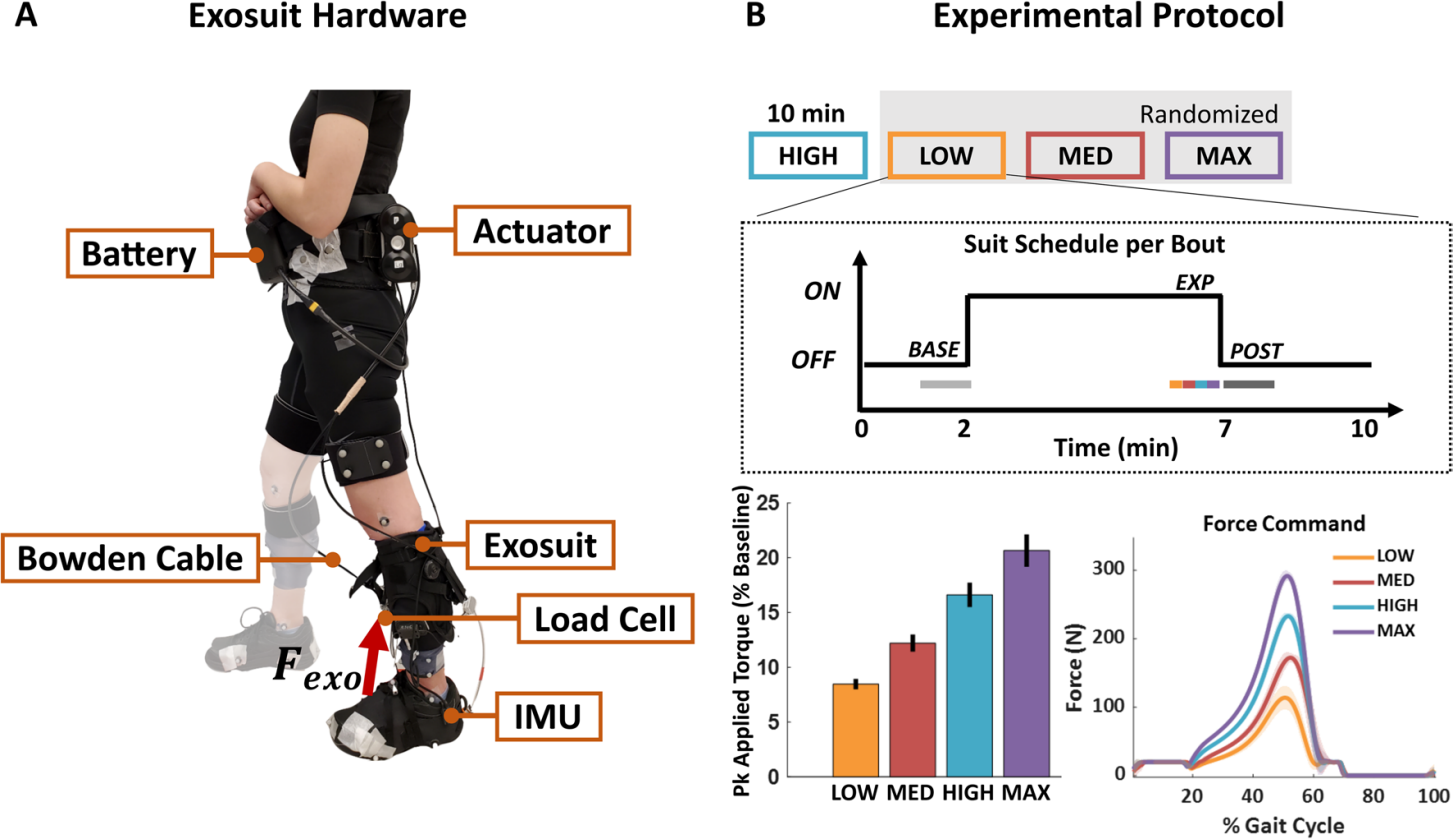
Experimental setup and protocol overview. **A** Unilateral soft ankle exosuit. **B** Experimental conditions for each subject and force application schedule for each condition (top). Applied peak torque for each condition across subjects normalized by peak biological ankle torque from an initial biomechanics collection without any device (bottom left). Applied force profile for each experimental condition across the gait cycle (0% is heel strike) for a sample subject (bottom right)

For this study, we used the soft medical exosuit for unilateral ankle assistance previously developed by our team (Fig. 1A, [39]). The fully autonomous exosuit delivered forces to resist ankle plantarflexion through a Bowden cable anchored to the individual using functional textile components. Specifically, the exosuit consisted of a calfwrap with the proximal anchor point for the cable at the anterior shin, and a custom-sewn sleeve wrapped around the shoe to provide the distal anchor point at the dorsal midfoot area of the shoe. A Fabrifoam® liner (Fabrifoam Products, Exton, PA, USA) was used to minimize drift of the calfwrap. A custom-designed waistbelt was used to mount the actuator unit. The total weight of all exosuit components including the actuator and battery was 4.1 kg, with approximately 3.6 kg located proximally at the waist, and the remaining distributed along the length of the limb. Further details on the base hardware design can be found in [39] and the component-wise weights are provided in Additional file 1: Fig. S1.

#### Controller

When in the active mode, on-board load cells (LSB200, Futek, Irvine, CA, USA) measured the tension in the cable, and inertial measurement units (IMUs) (MTi-3, XSens, Enschede, Netherlands) were used to identify gait events (heel strikes and toe-offs) for defining the force profile (Additional file 1: Fig. S2C, [24, 39]). The force controller used in this study built upon an admittance controller scheme introduced in previous exosuit work (Additional file 1: Fig. S2A, [24, 46]). Specifically, we implemented a nested force controller, with the inner loop running closed-loop control on the actuator motor velocity, and the outer loop containing a feedforward admittance term to account for exosuit compliance and a feedback term on the measured force.

The controller commanded a predetermined, subject-specific force profile to resist the user’s ankle that directly opposed the individual’s biological ankle torque, similar in principle to the approach in Conner et al. [27]. Each participant’s baseline ankle torque profile was determined in an initial biomechanics collection during which subjects walked on a treadmill without any device at 1.25 m s^−1^ for 2 min. We then performed offline IMU-based gait event detection to simulate controller state machine transitions, and time-normalized ankle torque profiles to span between estimated ipsilateral (resisted) heel strike and toe-off (Additional file 1: Fig. S2C). The subject-specific torque profile was then scaled by a measured cable moment arm to obtain the corresponding exosuit force profile. Constant moment arm was assumed throughout the stride and was measured as the perpendicular distance between the cable and the lateral malleolus while the subject was upright, and the cable was tensioned to 7.5 N. This force magnitude is a typical pretension level for this device to remove any excess length in the cable without applying appreciable force to the user.

When in the slack mode, the controller maintained a fixed cable position throughout the entire stride. The cable position was set such that the cable was not in tension throughout the gait cycle, and thus no forces were applied to the user (− 0.08 ± 0.76 N across all slack sections for all subjects). The cable to assist plantarflexion present on the exosuit by design was set to slack mode for the entirety of the experiment.

### Main experiment: experimental protocol

Each participant walked on a treadmill at 1.25 m s^−1^ for a series of four 10-min bouts, with each bout comprising 2 min of slack walking, followed by 5 min of active (resisted) walking, and 3 min of slack walking performed continuously in sequence (Fig. 1B). This speed falls within the range of comfortable walking speeds for healthy individuals and has been used in numerous assistive exoskeleton and exosuit studies [47]. For each bout, a researcher manually triggered the transition between active and slack to ensure participant safety and to prevent capturing a response to instability. Across the bouts, the commanded peak resistance force magnitudes were designed to correspond to 10, 15, 20 and 25% of peak biological ankle torque from the initial biomechanics collection, referred to as LOW, MED, HIGH, and MAX respectively (Fig. 1B). These magnitudes allowed us to study a broad range of resistance levels from barely perceptible to overpowering at the ankle, and were identified through internal testing. The HIGH condition was always administered first, while the order of the remaining three (LOW, MED, and MAX) was randomized. We opted to fix the first condition to both serve as a training bout and to measure the naïve response to ankle-targeted resistance across subjects at a consistent force level. One subject was unable to complete the MAX condition due to technical difficulties.

Standard instructions were provided, wherein participants were told to walk as “they normally would on a treadmill” or “as is most comfortable.” These bouts were conducted without explicit instructions as this has been reported to reflect the response of some poststroke subjects in other gait paradigms [48]. All subjects wore the device on the left leg, regardless of their leg dominance.

During the session, we collected optical motion capture data (Qualisys, Gothenburg, Sweden; 120 Hz) from both limbs and three-dimensional ground reaction forces (GRF) from an instrumented treadmill (Bertec, Columbus, OH, USA; 1200 Hz). Soleus muscle activity (SOL) was collected with surface electromyography (EMG) at 2040 Hz (Delsys, Natick, MA, USA). Exosuit sensor data from the on-board IMUs and load cells were streamed via Bluetooth at 100 Hz. We also collected subjective data after each walking bout, rating perceived fatigue and feasibility of the active section on a scale from 0 to 100. Subjects were given at least 2 min of rest between each bout and were allowed longer breaks if fatigued.

### Data analysis

#### Biomechanics

Motion capture and ground reaction force data were post-processed with a low-pass zero-phase filter with a 6 Hz cutoff to remove noise artifacts. We then computed inverse dynamics using these data to generate joint kinetics and kinematics with Visual3D software (C-Motion, Germantown, MD, USA). All kinetic variables were normalized by body mass. For EMG data, we first applied a fourth-order Butterworth bandpass filter from 20 to 450 Hz. The data were then rectified and low-pass filtered at 6 Hz to get the signal envelope. Finally, we normalized the data by subject and condition using the average peak value across all corresponding baseline strides. This approach reduced the effects of possible drift related to shifts in sensor location or changes in the skin-sensor interface across bouts. In the case of poor electrode connectivity leading to artifacts, the associated EMG data were excluded from further analysis. We segmented all data by gait cycle, using force plate data to detect heel strikes (0% gait cycle), and interpolated to 1001 points per stride. Strides in which the participant crossed belts on the treadmill were removed from analysis.

#### Suit

Force data from the exosuit were synchronized and combined with kinematic motion capture data to calculate exosuit and biological contributions to net ankle joint kinetics [49].

#### Sub-section definitions

For each subject, the last 60 s of each bout’s initial slack section, minute 1–2, were used as the baseline (BASE). We used the last 60 s of the active section, minute 6–7, to evaluate user response during exposure to resistance (EXP). Finally, the first 60 s of the post-active (POST) period, minute 7–8, were used to evaluate short-term retention after removal of resistance (Fig. 1B). One-minute subdivisions were chosen as this is a common timeframe for analysis in exosuit work [32, 50].

#### Metrics

*Ipsilateral plantarflexor effort* We used the peak ipsilateral biological ankle torque as an indicator of plantarflexor effort as it has been previously linked with modulating propulsion and gait speeds [5]. Biological torque was computed by taking the difference between the net torque obtained from inverse dynamics and the resistive torque applied by the exosuit [49]. We also computed the average activity of the soleus, a primary plantarflexor muscle, during the stance phase, i.e., heel-strike to toe-off for each stride, as a measure of one neuromotor mechanism through which biological torque can be modulated. While the medial gastrocnemius is another major plantarflexor muscle, its biarticulate structure also makes it a knee flexor. This dual functionality confounds analysis of its response to the resistive torques applied in this study, and thus we focused on changes in the soleus.

*Ipsilateral joint kinematics and kinetics (intralimb tradeoffs)* For each joint on the resisted limb, we computed the total positive or negative joint work done during stance by integrating the positive or negative joint power from heel-strike to toe-off, respectively. We also measured peak plantarflexion and dorsiflexion angle, peak knee flexion angle during mid-stance, and peak hip extension angle during stance.

*Bilateral limb loading (interlimb tradeoffs)* Limb loading was calculated as the average vertical ground reaction force during stance. By considering the average rather than the peak, we aimed to use a holistic measure of weight bearing performed by each limb throughout stance rather than a measure of impact.

### Statistics

*Primary* To evaluate the effects of resistance, a separate linear mixed-effects model was used at each force level to determine the effect of the different walking sections (BASE, EXP, and POST). For each comparison, the subjects were defined as random effects in the model and the walking sections as the repeating factor. Thus, the linear mixed-effects model allowed us to consider the participant population as heterogeneous while accounting for repeated measures in the protocol design. The dependent variables were peak ipsilateral ankle torque, average ipsilateral soleus activity, ipsilateral joint kinematics and joint work, and bilateral limb loading. Residuals of the data were checked to satisfy normality assumptions of the model. A separate linear mixed-effects model was used to evaluate the main effect of experimental order to determine whether conducting the HIGH condition first for all participants had a significant effect. We did not observe any order effects on the dependent variables (p > 0.05). For all variables, we also compared baselines across experimental conditions to ensure that effects from preceding conditions were fully washed out. All statistical analyses were run with custom MATLAB scripts (Mathworks, Natick, MA, USA).

*Secondary* In addition to the primary group-level analysis, we ran secondary, subject-level comparisons for evaluating the specificity of the applied resistance. As we expected variability in individual-level response due to the lack of explicit instructions, we anticipated that the force condition at which plantarflexor effort was maximized without engaging the unresisted joints would differ across subjects. Specifically, for each subject and condition, we evaluated whether limb loading in the strides from BASE and EXP were different from each other using a Mack-Skillings test at $\alpha$ = 0.05 significance levels. We used the tradeoff between plantarflexor effort and limb loading to indicate loss of specificity as our primary results suggested that interlimb tradeoffs precede intralimb tradeoffs.

As one subject was unable to complete the MAX condition due to technical difficulties and muscle activity data from some conditions were deemed unreliable, we report the final number of subjects used for each condition and variable alongside the statistical results in the corresponding text and figures.

### Exploratory sub-studies: experimental protocol

For two individuals (1M, 1F; age = 28.5 ± 3.5 years (mean ± std), mass = 56 ± 2.8 kg, height = 1.67 ± 0.02 m), we conducted two sub-studies on a separate day to investigate efficacy of the resistive exosuit paradigm for joint and phase-specific training. We compared the task-specificity of the resistive exosuit against a conventional resistance training method of applying a passive force at the pelvis with a resistance band. We also evaluated the effect of providing explicit instructions on the targeted ankle plantarflexor kinetics. A single force level (MED) was used in these exploratory pilots as a preliminary investigation. We collected the full set of measurements from the main experiment during both exploratory collections. Only descriptive statistics for basic features of the data (e.g., mean) were evaluated given the small sample size. The two individuals had participated in the main experiment, but because the gap between these two experiments was over two weeks for each subject, we assumed that training was not a factor [51].

#### Resistance band

Subjects walked for two 10-min bouts at 1.25 m s^−1^ using the same subdivisions as in the main experiment, once with the MED exosuit-applied resistance condition (EXO) and once with a resistance band fitted with a custom load cell (BAND) similar to Lewek et al. [10]. We positioned the band to apply a constant force of approximately 10% body weight (BW) during the exposure section, using the maximum force reported in Lewek et al. as a benchmark [10]. Subjects were instructed to walk as was most comfortable on the treadmill during both, EXO and BAND bouts, similar to the main experiment. We evaluated changes in peak biological ankle torque, peak torso angle, peak hip torque, and average biological ankle torque in the early (0–20 %GC), mid (20–40 %GC), and late stance (40–65 %GC) phases.

#### Explicit Instructions

Subjects walked for two 10-min bouts at 1.25 m s^−1^ in the MED resistance condition using the same subdivisions as in the main experiment. The same implicit instructions from the main experiment to walk as was most comfortable were again used during the first condition (Implicit). In the second condition (Explicit), subjects were given explicit instructions to pushoff against the force and resist the exosuit. Implicit and explicit instructions thus differed by whether they were designed to encourage voluntary effort from the participant to resist the applied force. The order was not randomized so that the explicit instructions would not influence the implicit response. We evaluated changes in plantarflexor effort and limb loading.

## Results

### Exosuit performance

The average applied peak exosuit torques across force conditions were 0.13 ± 0.01 N m kg^−1^ (mean ± s.e.m) for LOW, 0.19 ± 0.01 N m kg^−1^ for MED, 0.26 ± 0.02 N m kg^−1^ for HIGH, and 0.32 ± 0.02 N m kg^−1^ for MAX. Across subjects, these torques correspond to approximately 8.5%, 12.2%, 16.6%, and 20.7% of peak ankle torque from the initial biomechanics collections (Fig. 1B). The average applied peak exosuit forces across conditions were 124.4 ± 5.5 N (mean ± s.e.m) for LOW, 186.7 ± 8.4 N for MED, 247.2 ± 11.1 N for HIGH, and 313.4 ± 13.0 N for MAX. These forces correspond to approximately 19.0%, 28.4%, 37.7%, and 46.7% BW. The RMSE of peak applied force during the active sections of each trial across all subjects and conditions was 5.36 ± 2.70 N (mean ± std). The standard deviation in peak applied force per trial across all subjects and conditions was 4.04 ± 1.27 N (mean ± std). Although force tracking performance was similar to prior exosuit work [24, 52], peak exosuit torques were lower than designed at each resistance level. This discrepancy suggests that the dynamic cable moment arm, obtained through post-processing, was lower than the static estimate used to define the applied force profiles.

### Effects of exosuit resistance on ipsilateral plantarflexor effort during EXP

**Fig. 2:**
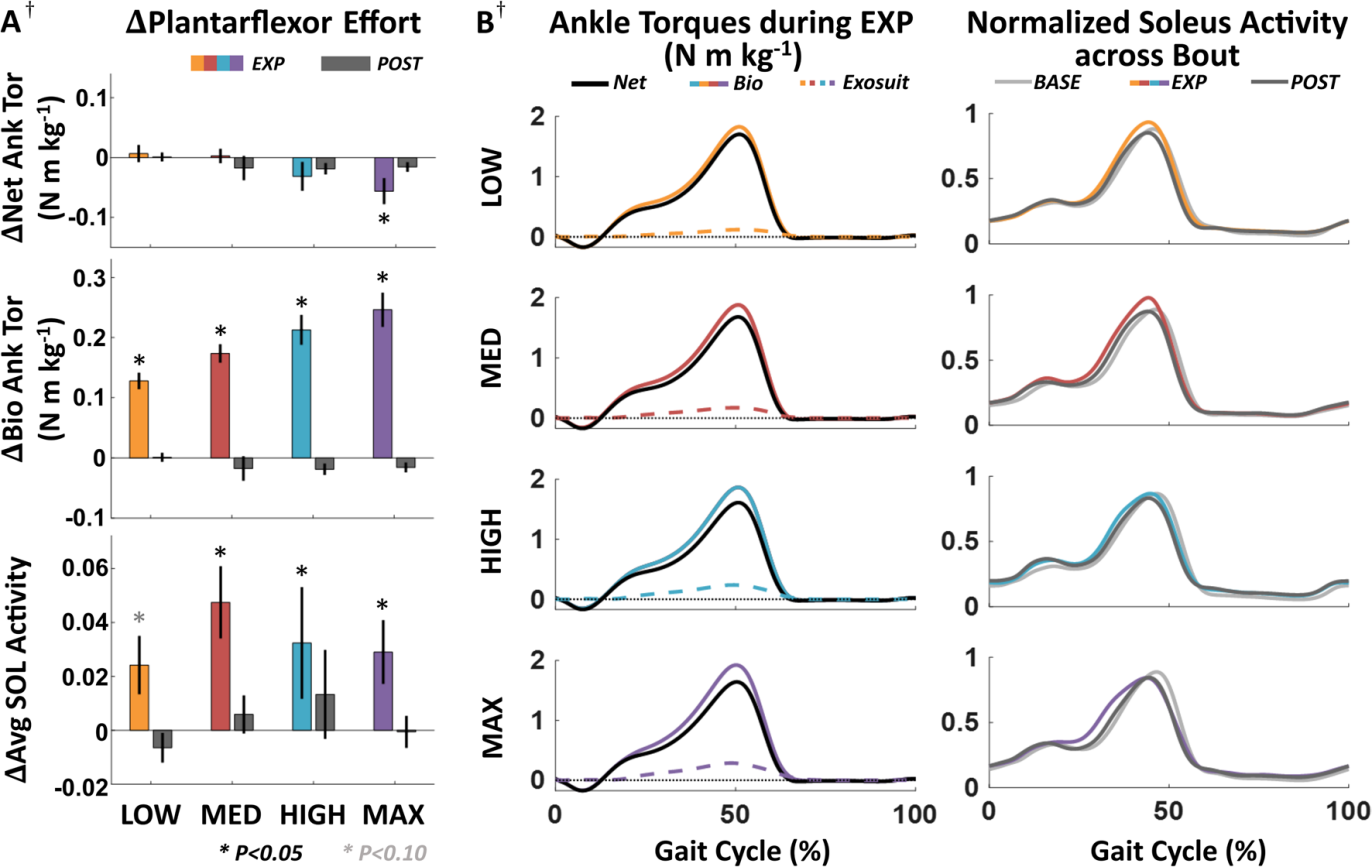
Ipsilateral plantarflexor effort across resistance magnitudes. **A** Change in peak net and biological ankle torque, and normalized soleus activity during stance during EXP and POST relative to BASE across all subjects. **B** Average ankle torque profiles during EXP across all subjects for each condition, normalized by body mass (left). Exosuit torque magnitude is plotted here for figure space efficiency but is negative for all conditions and subjects (see Additional file 1: Fig. S2C). Average normalized soleus activation profiles across all subjects for each condition during BASE, EXP, and POST (right). ^†^One subject did not complete the MAX condition, and one subject did not have usable EMG data from the MED and HIGH conditions (N = 9). All error bars are s.e.m

Peak biological ankle torque increased significantly to negate the exosuit-applied resistance force during EXP compared to BASE across all resistance force levels (p < 0.001) (Table 1, Fig. 2). Significant increases were seen in average soleus activity during stance at the MED, HIGH and MAX conditions (p < 0.05), while there was a trend towards increased activity at the LOW condition (p = 0.090). Plantarflexor effort during BASE did not differ across conditions (p > 0.10).

**Table 1:** Changes in ipsilateral plantarflexor kinetics and muscle activity during EXP

Variable	Resistance Condition							
	LOW		MED		HIGH		MAX	
	Change	p-value	Change	p-value	Change	p-value	Change	p-value
Peak net ankle torque (N m kg^−1^)	0.01 ± 0.01	0.793	0.00 ± 0.01	0.918	− 0.03 ± 0.02	0.212	− 0.06 ± 0.02^†^	**0.036**
Peak bio ankle torque (N m kg^−1^)	0.13 ± 0.01	** < 0.001**	0.18 ± 0.02	** < 0.001**	0.21 ± 0.02	** < 0.001**	0.25 ± 0.03^†^	** < 0.001**
Positive bio ankle work (J kg^−1^)	0.029 ± 0.010	**0.036**	0.034 ± 0.008	**0.013**	0.036 ± 0.009	**0.004**	0.053 ± 0.015^†^	** < 0.001**
Avg soleus stance activation (normalized)	0.024 ± 0.011	0.090	0.047 ± 0.013^†^	**0.001**	0.032 ± 0.021^†^	**0.022**	0.029 ± 0.012^†^	**0.046**

Data are mean ± s.e.m. Each value is the difference between EXP and BASE for the corresponding condition and subject. Bold values indicate significance (p < 0.05)

^†^N = 9, otherwise N = 10

### Effects of exosuit resistance on ipsilateral joint kinematics and work during EXP

**Fig. 3:**
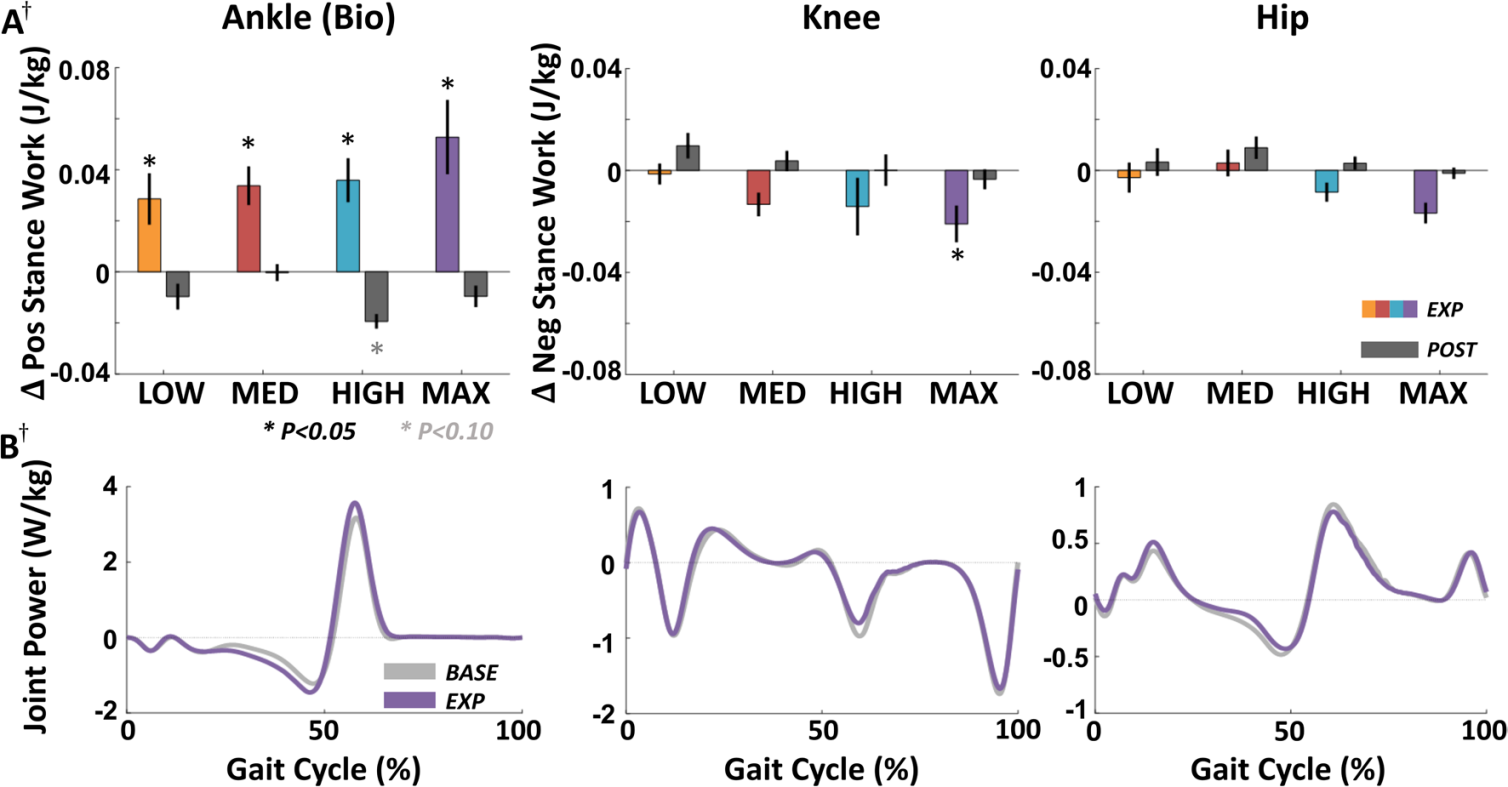
Ipsilateral joint work across resistance magnitudes. **A** Changes in magnitude of positive biological ankle work during stance (left), and magnitudes of negative knee and hip work during stance (center, right) in EXP and POST relative to BASE. **B** Average ankle, knee, and hip joint power profiles across the gait cycle in the MAX condition across all subjects. ^†^One subject did not complete the MAX condition (N = 9). All error bars are s.e.m

Like biological torque, positive biological ankle work during stance increased significantly during EXP compared to BASE across all force magnitudes (p < 0.05) (Table 1, Fig. 3). We also found that peak ankle plantarflexion angle decreased by 2 to 4 deg (Additional file 1: Table S1, Fig. S3) across all conditions (p < 0.05). However, while ankle kinetics and kinematics changed across all conditions, we observed changes at the ipsilateral knee and hip joints only at the highest force levels. Knee flexion angle in mid-stance increased significantly in the HIGH and MAX conditions (p < 0.05), corresponding to increases in dorsiflexion angle (p < 0.05) and decreases in peak hip extension angle that trended to significance (p < 0.10).

Moreover, the magnitude of negative work at the ipsilateral knee during stance decreased at the MAX resistance level by 0.021 ± 0.007 J kg^−1^ (p = 0.026, n = 9), and non-significantly at the HIGH resistance level by 0.014 ± 0.011 J kg^−1^ (p = 0.111, n = 10) (Fig. 3). This change was driven by the increased knee flexion angle during the mid-late stance leading to reduced knee flexion velocity during pushoff, which translated to decreased negative knee power, and thereby negative knee work. Across all conditions, negative work done during stance at the ipsilateral hip did not change significantly (p > 0.10).

Ipsilateral joint kinematics and work measures during BASE did not differ across conditions (p > 0.10).

### Effects of exosuit resistance on bilateral limb loading during EXP

**Fig. 4:**
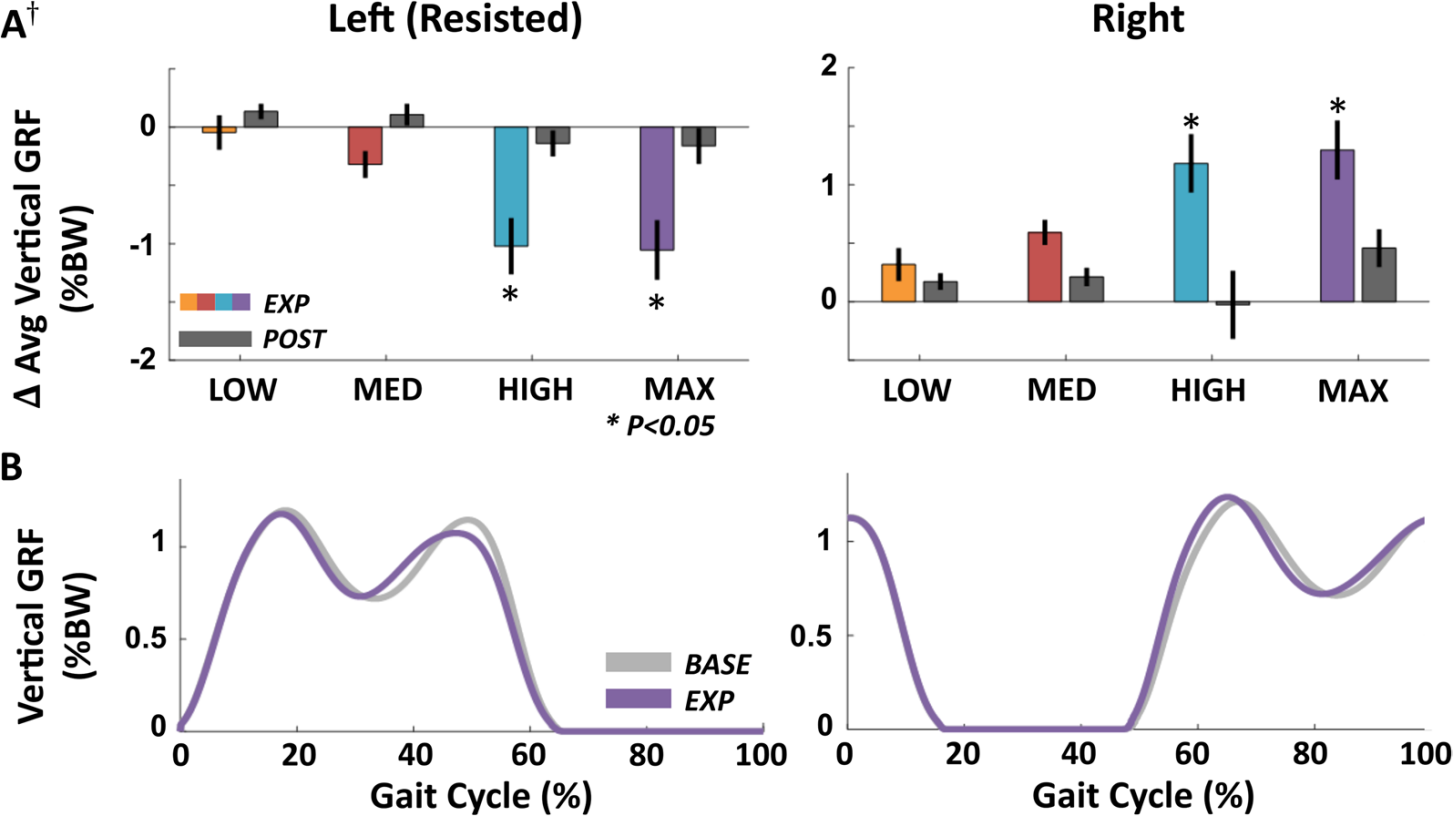
Average vertical ground reaction forces across resistance force magnitudes. **A** Change in resisted ipsilateral (left) and unresisted contralateral (right) average vertical ground reaction force during stance in EXP and POST relative to BASE across all subjects. **B** Vertical ground reaction force during BASE and EXP for a single subject at the HIGH condition segmented by ipsilateral heel strikes. †One subject did not complete the MAX condition (N = 9). All error bars are s.e.m

As hypothesized, we found that at high applied resistance magnitudes, limb loading on the resisted ipsilateral limb decreased, while increasing on the unresisted contralateral limb. Specifically, at the LOW and MED resistance magnitudes, ipsilateral average vertical ground reaction forces were unchanged (p > 0.10, n = 10), but decreased by 1.02 ± 0.24 %BW (p = 0.002, n = 10), and 1.05 ± 0.26 %BW (p = 0.005, n = 9) at the HIGH and MAX force levels, respectively (Fig. 4). We observed corresponding increases in average contralateral vertical ground reaction forces of 1.18 ± 0.25 %BW (p < 0.001, n = 10) and 1.30 ± 0.25 %BW (p < 0.001, n = 9) at the HIGH and MAX force levels, but not at the LOW or MED force levels (p > 0.10). Limb loading on both legs during BASE did not differ across conditions (p > 0.10).

### Effects of exosuit resistance on gait biomechanics during POST

**Fig. 5:**
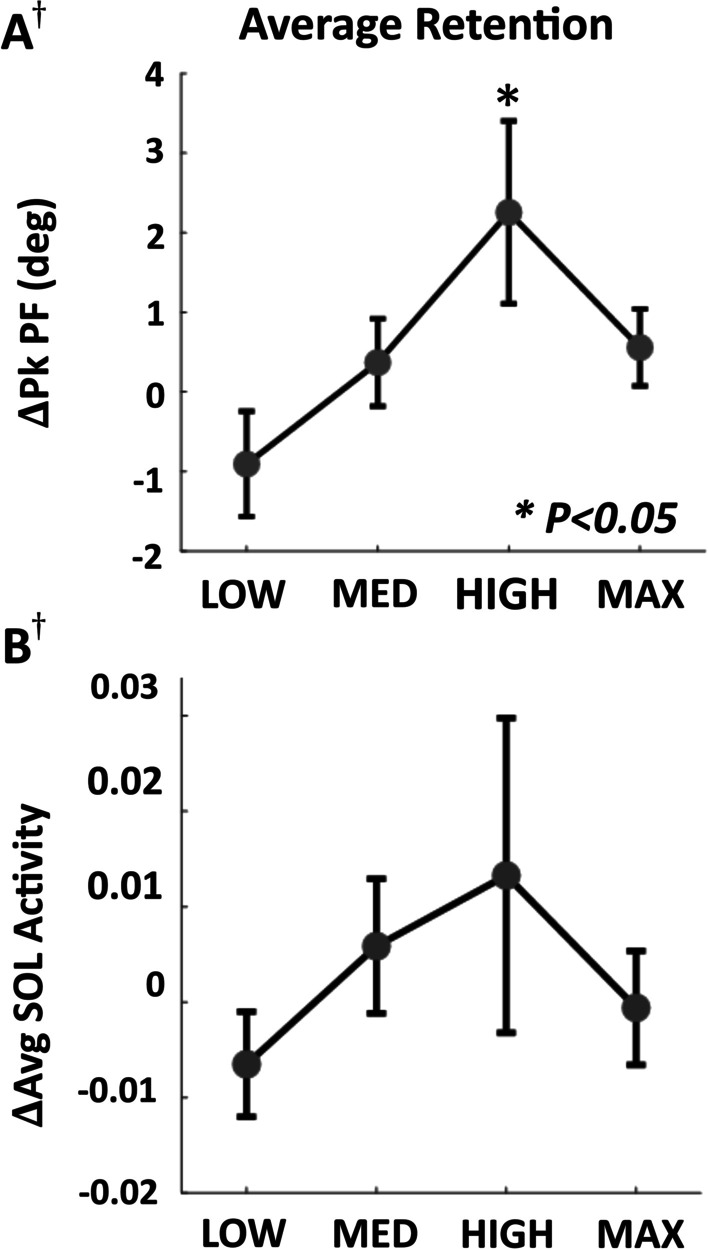
After-effects upon removal of resistance at each force magnitude. **A** Change in peak plantarflexion angle during POST compared to BASE. **B** Change in mean soleus (SOL) activity during stance during POST compared to BASE. ^†^One subject did not complete the MAX condition and one subject did not have usable SOL data for MED and HIGH (N = 9). All error bars are s.e.m

At the group level, post-active peak plantarflexion angle increased by 2.5 ± 1.1 deg (p = 0.017, n = 10) at the HIGH force magnitude (Fig. 5A). Order did not have a significant main effect for this variable (p = 0.728, n = 10). Although the increase in average soleus activity during POST was also greatest in the HIGH force magnitude at 0.013 ± 0.017 (normalized), changes were insignificant (p = 0.320, n = 9) (Fig. 5B, Additional file 1: Table S2). No significant changes were observed during POST in other outcome variables at any force level (p > 0.05).

### Individual-level analysis of plantarflexor effort and limb loading during EXP across resistance magnitudes

**Fig. 6:**
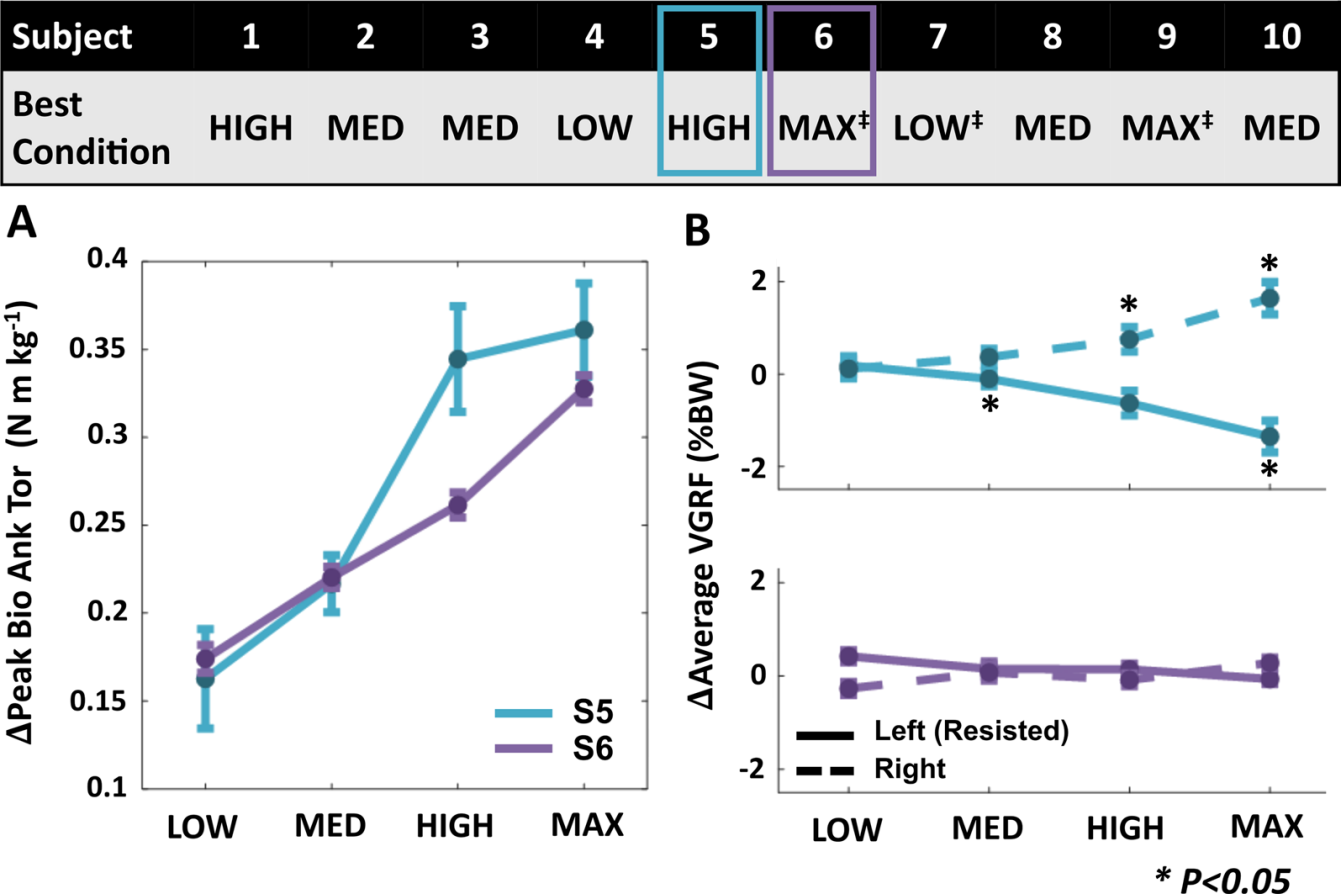
Effects of resistance magnitude on specificity during EXP at an individual level (Table 1). Condition that maximizes increase in peak biological ankle torque without changes in bilateral limb loading for each subject. A shift in limb loading is considered significant if both the left and right limbs change significantly. **A** Representative changes in peak biological ankle torque data from two subjects. **B** Changes in average stance vertical ground reaction force on both limbs. ^‡^The resistance magnitude that maximally targets the ankle may lie outside the explored range for these subjects. All error bars are s.e.m

Each subject increased peak biological ankle torque relative to BASE at each force magnitude (p < 0.001), with most individuals showing greater increases at higher resistance levels. However, the conditions with significant shifts in limb loading, i.e., reduced loading on the ipsilateral limb accompanied by increased loading on the contralateral limb, differed across individuals. Consequently, the force magnitude at which plantarflexor effort was maximized without incurring overreliance on the contralateral limb also varied (Fig. 6). Across our subjects, specificity of the applied resistance to the ipsilateral ankle was greatest in the LOW condition for 2 subjects, the MED condition for 4 subjects, the HIGH condition for 2 subjects, and the MAX condition for 2 subjects.

### Exploratory: comparison between resistive exosuit and conventional resistive band training

**Fig. 7:**
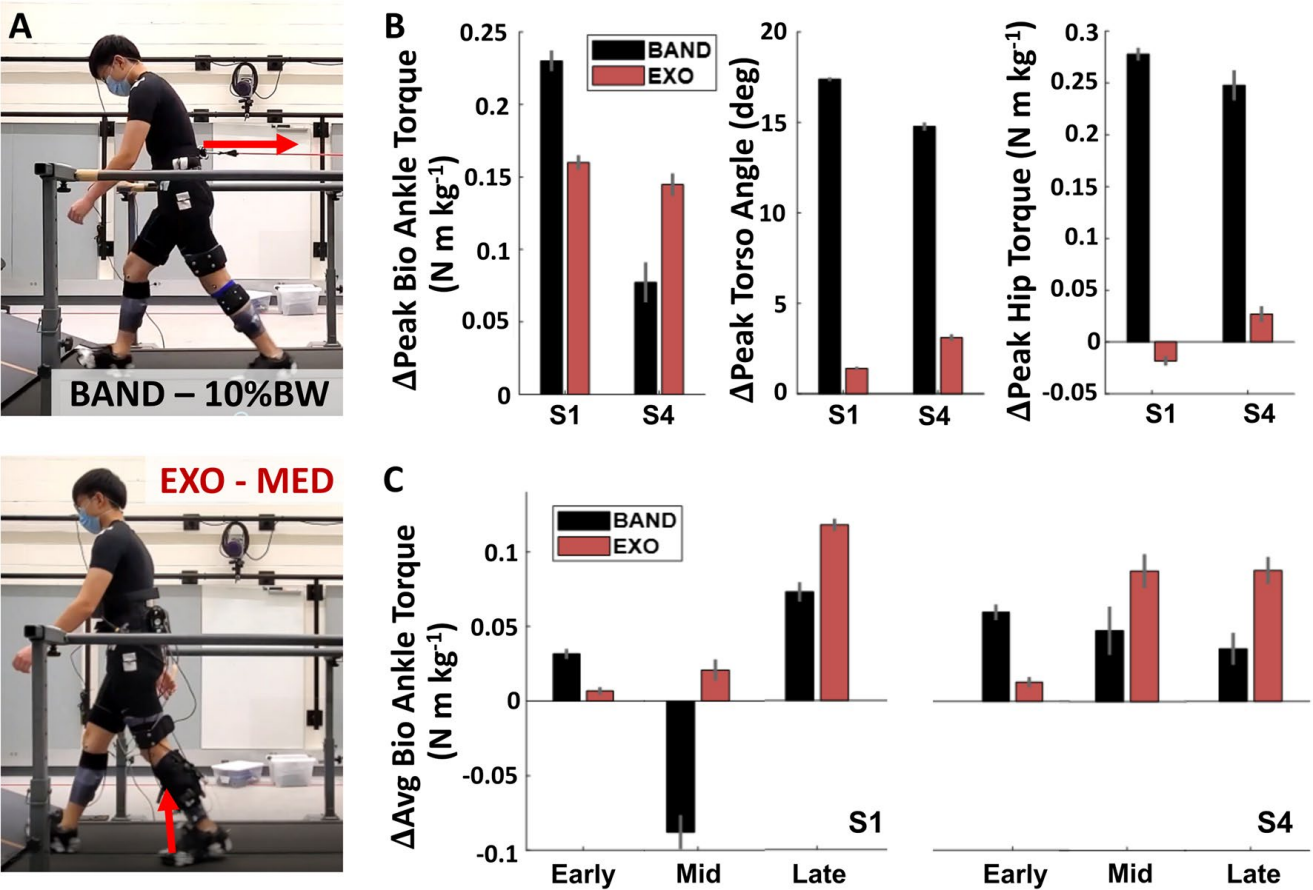
Comparison with conventional resistive band training during EXP. **A** Participant walking with passive resistance from a sensorized resistance band (top) and with exosuit-applied plantarflexion resistance (bottom). **B** Changes in peak biological ankle torque, peak torso angle, and peak hip torque in early stance with respect to BASE for both subjects (S1 and S4). **C** Changes in average biological torque during the early (0–20 %GC), mid (20–40 %GC), and late (40–65 %GC) stance phase with the band and exosuit applied resistance conditions

Regardless of training paradigm, both subjects increased peak biological ankle torque during EXP compared to BASE (Fig. 7, Additional file 1: Table S3). Both subjects demonstrated greater increases in average biological ankle torque during early stance with the resistance band, and greater increases during mid-late stance with the resistive exosuit. Peak hip torque and peak torso angle also increased more with the resistance band.

### Exploratory: effect of explicit instructions during EXP

**Fig. 8:**
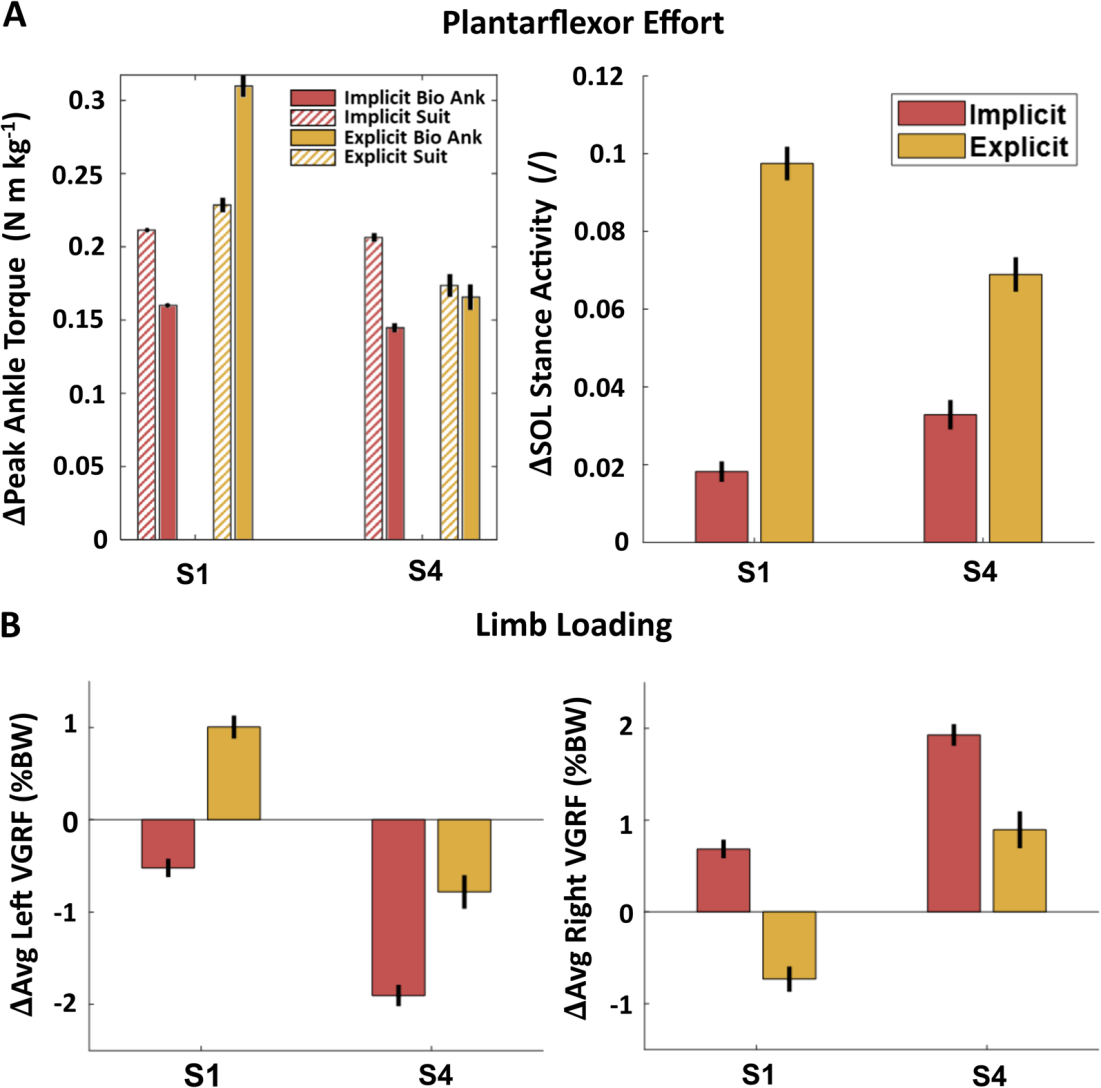
Effects of explicit instructions during EXP. **A** Change in peak biological ankle torque compared to peak applied exosuit resistive torque (left). Change in average soleus (SOL) activity (right). **B** Change in average vertical ground reaction force (VGRF) on ipsilateral (left) and contralateral (right) limbs with implicit and explicit instructions for both subjects

Both subjects demonstrated greater increases in peak biological ankle torque and soleus activity relative to BASE with explicit instructions (Fig. 8A, Additional file 1: Table S4). These changes were accompanied by reduced shifts in limb loading as measured by smaller decreases in ipsilateral average vertical ground reaction forces and smaller increases on the contralateral limb (Fig. 8B).

## Discussion

This study aimed to investigate the user’s biomechanical response to an exosuit that resists ankle plantarflexion during stance, specifically testing the hypothesis that plantarflexor kinetic and muscle activity would increase with increasing active resistance. This is the first time, to our knowledge, that the effects of joint-targeting resistance magnitude on healthy gait have been studied. We demonstrated that the applied resistance increased plantarflexor effort in the resisted joint across all applied force magnitudes through increases in peak biological ankle torque and average soleus stance activity, with larger average increases in biological torque at higher levels of resistance. However, we found that participants also increased reliance on the unresisted limb with average vertical ground reaction force during stance decreasing on the ipsilateral limb and increasing on the contralateral limb at the two highest resistance magnitudes. Moreover, at the highest force level, participants showed small but significant changes in the work done by the ipsilateral knee during stance, further highlighting the importance of the applied resistance level on the resulting gait response.

The results of this study support the recent findings from others [27, 35, 53], that locally applied resistance (to oppose biological torque) at the ankle can increase plantarflexor kinetic and muscle activity. Furthermore, the average increase in peak torque was larger than the reported minimal detectable change (MDC) of 0.13–0.16 N m kg^−1^ during overground walking in the MED to MAX force conditions [54]. The MDC has been used to determine clinical significance for changes in poststroke gait, and thus these results suggest the feasibility of the resistive exosuit paradigm for increasing plantarflexor effort in people poststroke. Another recent study found that in the presence of plantarflexor resistance, healthy individuals reduced ankle joint range of motion and peak plantarflexion angle [32]. While our findings are consistent with these outcomes, we observed only slight reductions in peak plantarflexion angle (2–4°) when applying up to 300 N of force compared to the approximately 10° seen in prior work when applying 10 N [32]. The changes in our study are within the previously reported MDC of 3.97 to 4.55° for healthy individuals walking overground at different speeds [54]. Unlike the approach in [32], we selectively targeted the stance phase, and therefore ramped down the resistive force prior to the onset of swing. Thus, our results highlight the importance of targeting the stance phase to ensure the ankle is less kinematically constrained, an important consideration for the goal of increasing walking speeds in clinical applications [55].

Despite consistent increases in peak ipsilateral biological ankle torque with increasing resistance force magnitude, this systematic upregulation was not observed in plantarflexor muscle activity, indicating that some of the changes in torque were driven by other mechanisms. We expected to observe intralimb and interlimb tradeoffs in which the participant increased reliance upon the unresisted joints and limb, given previous work that found a redistribution of kinetics across limbs in high intensity tasks [20, 38]. At the intralimb level, we found that users decreased negative work at the ipsilateral knee at the highest resistance level, suggesting that users succumbed to the applied resistance at these magnitudes. These findings are consistent with analogous work in which people poststroke reduced negative knee work while walking with an ankle–foot orthosis with high torsional stiffness that resisted plantarflexion [56]. Our results also mirror the conclusions from a review of ankle–foot orthosis designs and their effects on poststroke gait, which reported changes in proximal kinetics [57]. At the interlimb level, we observed that with increasing resistance magnitude, participants decreased the average vertical ground reaction force during the stance phase on the ipsilateral limb with an associated increase on the contralateral limb. This weight shift pattern may be similar to that of people poststroke who often show more limb loading on the non-paretic side [58] due to reduced net efficiency of the paretic joint. We found that participants exhibited changes in limb loading prior to changing ipsilateral joint kinetics, suggesting that the response to high loads at the ankle may occur primarily at the interlimb level, and secondarily at the intralimb level. Overall, these findings suggest that there is a point at which the subject’s strategy shifts towards using the unresisted limb rather than the resisted joint, resulting in the observed discrepancy between biological torque and muscle activity with increasing resistance. Subjective feedback also reflected this result as participants reported increased fatigue and reduced feasibility at higher resistance force magnitudes (Additional file 1: Fig. S4). Thus, a holistic quantification of the response to ankle-targeting resistance across varying magnitudes will require multidimensional analyses that consider both the resisted and unresisted joints.

Further examination of individual-level response showed that across the participant cohort, different subject-specific resistance magnitudes were needed to best target the resisted ankle joint without engaging the unresisted limb. We expected that to maximize targeting the resisted ankle joint, the resistance level would need to be individualized to account for subject-specific variability in physiology. We found that overall, the MED condition offered greatest specificity of the applied resistance to the ankle. However, the LOW, HIGH, and MAX conditions were also determined to maximize specificity for certain participants. One subject exhibited a shift in limb loading even at the LOW condition, and thereby may benefit from exploring lower resistance magnitudes. Conversely, two subjects were able to walk at the MAX condition without significant shifts in limb loading, and thus may be able to tolerate even higher resistance levels. This variability is consistent with the well-documented importance of individualization for assistive robotic devices, which has been shown to influence improvements in energetic cost in healthy individuals [25, 59, 60] and changes in joint kinetics in stroke survivors [24, 50]. Moreover, the importance of varying resistance force magnitude for optimizing motor performance aligns with the challenge point theory framework, which posits the existence of an optimal, subject-specific task intensity for maximizing motor learning outcomes [61, 62]. Given that unimpaired individuals demonstrate sensitivity to force levels, we anticipate that resistance magnitude will be also an important parameter setting for applications in people poststroke given their complex neuromotor profiles. Our findings suggest that using changes in bilateral limb loading and biological ankle torque may enable effective individualization of joint-targeting resistance magnitudes that improve exosuit-based training for specifically increasing plantarflexor effort.

We also hypothesized that participants would present with increased soleus activity relative to baseline when the exosuit resistance was removed as short-term retention, and that this increased activity would result in increased peak plantarflexion angle [63, 64]. Although we did not find significant retention in soleus activity for any force level, the applied plantarflexion resistance led to significant short-term after-effects in peak plantarflexion angle at the HIGH condition. This is consistent with a recent study that showed that walking overground with targeted passive resistance at the ankle and explicit instructions to pushoff against the force led to increased plantarflexion angle in the first 5 strides after doffing the resistive element [33]. Our results indicate that in the absence of explicit instructions, these after-effects can be observed when at an appropriate intermediate resistance magnitude through an even longer time window of one minute. Furthermore, despite the statistical insignificance of group-level retention in soleus activity, the magnitude of average retention of soleus activity aligned with the magnitude of the after-effect in peak plantarflexion angle. These findings may shed light on how targeted joint level resistance can be applied during training to increase plantarflexor effort during post-active gait. Similar to the need for subject-specific force magnitudes to maximize specificity to the ankle, this result suggests that there may also be optimal resistance force magnitudes that maximize the carryover of increased soleus activity induced by the resistance force into gait immediately after removal of resistance.

The proposed method of applying targeted resistance with an exosuit may further enable increased joint and phase specificity during training compared to the conventional global method of using passive resistive bands that apply a force at the pelvis opposing the direction of motion [10]. In our exploratory pilot with two subjects, we found that while both methods increased peak biological ankle torque, the band also induced large changes in peak torso lean and peak ipsilateral hip torque, thereby resulting in a whole-body response to the global resistance, similar to predictions from a recent simulation study [14]. Moreover, the band altered ankle torque throughout the entire stride, with the largest changes during early-stance, while the exosuit resulted in maximal changes during mid-late stance. Thus, for these two subjects, the exosuit applied more targeted and functional resistance specific to increasing ankle kinetics during the mid-late stance phase, a region of interest for poststroke gait rehabilitation.

We found that the instruction given to participants during exosuit-applied plantarflexion resistance training also played an important role in targeting the plantarflexors. Although providing no explicit instructions enabled us to capture the natural tradeoff between intralimb and interlimb changes in kinetics, the importance of task-specific instructions is well recognized both in the exoskeleton [65] and rehabilitation fields [66, 67]. In the two-subject exploratory study, participants generated sufficient biological ankle torque to match or overcome the applied torque when instructed to pushoff against the exosuit-applied resistance, while only partially offsetting the applied torque when uninstructed. The increase in ankle torque with explicit instruction further led to less reliance on the contralateral limb, with smaller reductions in ipsilateral ground reaction forces and smaller increases on the contralateral limb. Although only in a sample of two subjects, this finding suggests that with explicit instructions we may be able to shift the onset of contralateral limb engagement to higher resistance levels, enabling increased intensity that continues to target the resisted joint. Future work may consider repeating these procedures with explicit instructions either verbally or through visual feedback to systematically evaluate whether the optimal resistance level is shifted for each subject and if inter-subject variability is reduced. Moreover, for a clinical user group, explicit instructions will likely be an important factor for increasing plantarflexor effort and better targeting the impaired ankle [68].

Our findings demonstrate that a unilateral soft resistive ankle exosuit can selectively increase plantarflexor effort in the resisted joint during the stance phase. We further have shown the role of resistance magnitude as a parameter that modulates specificity. Finally, our results provide initial indicators of the value of the paradigm over a typical clinical resistive approach. Still, there are a few considerations that future work must account for prior to clinical translation. The force profiles used in this study were defined using the subject’s baseline ankle torque pattern which necessitated generating the profiles at a specific fixed speed and conducting all experimental conditions at this speed. However, for a healthy individual without existing gait asymmetries, any increases in unilateral plantarflexor effort must be negated elsewhere to prevent acceleration along the treadmill, which inherently limited the magnitude of change during and immediately after active resistance we could observe. We also assumed that our subjects were symmetric and did not account for leg dominance, similar to other recent investigations of ankle-targeting resistance in healthy populations [33]. Yet people poststroke have slower gait speeds and exhibit more asymmetry than age-matched unimpaired individuals, and furthermore, are often categorized by walking speed [69]. Thus, while we expect device performance and general trends to be consistent at slower speeds, this study cannot guarantee that the response in poststroke populations will directly conform to the findings we have presented here. The exosuit has the inherent ability to provide both assistance and resistance, in both constrained lab environments and unconstrained overground and community settings. This added versatility enables increased task-specificity on top of joint and phase specificity, another important factor for clinical gait rehabilitation efficacy [11], and is not fully explored in this study. We expect that future work towards individualized ankle-targeted exosuit resistance will investigate characterizing the poststroke gait response, alternative methods of defining resistance profiles, and integrating mobile sensing for variable speeds and environments.

## Conclusion

This paper presents the first study to systematically vary the magnitude of targeted active resistance and characterize the gait response across both lower limbs. Through this work, we aimed to generate fundamental understanding on the unimpaired response to ankle plantarflexion resistance with an exosuit across a range of force levels to inform its future application in poststroke gait rehabilitation. As expected, we found that biological ankle torque and soleus muscle activity increased at all resistance force levels. Furthermore, we showed that in the absence of explicit instructions and at the highest applied resistance magnitudes, individuals increased reliance on the unresisted contralateral limb and ipsilateral proximal joints. These tradeoffs were negligible at lower forces, suggesting that with an appropriate force level, we can target the resisted joint more exclusively. Further investigation showed that the force level that maximized targeting the resisted ankle without involving the unresisted limb varied across subjects. We also demonstrated that peak plantarflexor angle increased relative to baseline upon removal of resistance, but only at an intermediate force magnitude. Finally, this study generated initial insights to the effects of the form of resistance and the provided instructions through two exploratory pilots, which suggested that the ankle exosuit could better provide ankle-targeted resistance compared to an elastic band at the pelvis, and that explicit instructions to pushoff against the resistance could further increase specificity to the plantarflexor muscle. These results motivate the investigation of exosuit-applied plantarflexion resistance for training increased plantarflexor effort during mid-late stance for individuals poststroke.

## Supplementary Information

**Additional file 1: Fig. S1.** Exosuit component weights. Component-wise distribution of mass along the user’s body. Masses of textile components (waistbelt and calfwrap) are provided for the medium sizes, which were used by most participants. **Fig. S2.** Force controller architecture and performance. (A) Controller architecture with PI force feedback and admittance feedforward terms to command motor position and velocity to the actuator. (B) Average applied peak exosuit torque across all subjects for each condition (left). Average applied torque profiles across the gait cycle across all subjects for each condition (right). (C) Tracking performance for an example stride (top). Resultant exosuit-applied torque and baseline ankle torque for one subject (bottom). **Fig. S3.** Joint kinematic response across magnitudes. (A) Average change in peak dorsiflexion and plantarflexion angle, knee flexion angle during mid-late stance (20-65 %GC), and peak hip extension angle during EXP and POST relative to BASE across all subjects. (B) Averaged joint kinematics across all subjects for each condition and section plotted against the gait cycle (0% is heel strike). Local extrema within each boxed region used to generate bar plots in Panel (A). † One subject did not complete the MAX condition (N = 9). All error bars are s.e.m. **Fig. S4.** Subjective survey data. Average feasibility and fatigue scores across all subjects in each condition. Feasibility scores indicate the projected ability to walk with the active resistance for 15min continuously, where 0 is impossible and 100 is no foreseeable concern. Fatigue scores indicate the level of fatigue after the active resistance, where 0 is no fatigue. Feasibility relates to comfort while fatigue relates to muscle soreness. †One subject did not complete the MAX condition (N = 9). All error bars are s.e.m. **Table S1.** Changes in ipsilateral joint kinematics during EXP. **Table S2.** Effects of exosuit resistance on gait during POST. **Table S3.** Comparison of a passive resistance band and resistive exosuit in a two-subject pilot. **Table S4.** Effect of instructions on ankle plantarflexor effort in a two-subject pilot.

## Data Availability

The datasets used and/or analyzed during the current study are available from the corresponding author on reasonable request.

## Abbreviations

IMU: Inertial measurement unit
BASE: Baseline
EXP: Exposure to resistance
POST: Post-exposure to resistance
EMG: Electromyography
SOL: Soleus
VGRF: Vertical ground reaction force
GC: Gait cycle
SEM: Standard error of the mean
BW: Body weight
MDC: Minimal detectable change
