# Supplementary material for: Ankle resistance with a unilateral soft exosuit increases plantarflexor effort during pushoff in unimpaired individuals
Source: J Neuroeng Rehabil. 2021 Dec 27;18:182. doi: 10.1186/s12984-021-00966-5 (PMC8711150; doi:10.1186/s12984-021-00966-5)
Supplement: Supplementary file 1 — Additional file 1: Fig. S1. Exosuit component weights. Component-wise distribution of mass along the user’s body. Masses of textile components (waistbelt and calfwrap) are provided for the medium sizes, which were used by most participants. Fig. S2. Force controller architecture and performance. (A) Controller architecture with PI force feedback and admittance feedforward terms to command motor position and velocity to the actuator. (B) Average applied peak exosuit torque across all subjects for each condition (left). Average applied torque profiles across the gait cycle across all subjects for each condition (right). (C) Tracking performance for an example stride (top). Resultant exosuit-applied torque and baseline ankle torque for one subject (bottom). Fig. S3. Joint kinematic response across magnitudes. (A) Average change in peak dorsiflexion and plantarflexion angle, knee flexion angle during mid-late stance (20-65 %GC), and peak hip extension angle during EXP and POST relative to BASE across all subjects. (B) Averaged joint kinematics across all subjects for each condition and section plotted against the gait cycle (0% is heel strike). Local extrema within each boxed region used to generate bar plots in Panel (A). † One subject did not complete the MAX condition (N = 9). All error bars are s.e.m. Fig. S4. Subjective survey data. Average feasibility and fatigue scores across all subjects in each condition. Feasibility scores indicate the projected ability to walk with the active resistance for 15min continuously, where 0 is impossible and 100 is no foreseeable concern. Fatigue scores indicate the level of fatigue after the active resistance, where 0 is no fatigue. Feasibility relates to comfort while fatigue relates to muscle soreness. †One subject did not complete the MAX condition (N = 9). All error bars are s.e.m. Table S1. Changes in ipsilateral joint kinematics during EXP. Table S2. Effects of exosuit resistance on gait during POST. Table S3. Comparison of [file 12984_2021_966_MOESM1_ESM.docx]

**Ankle resistance with a unilateral soft exosuit increases plantarflexor effort during pushoff in unimpaired individuals - Supplemental Material**

Krithika Swaminathan^1^*, Sungwoo Park^1^*, Fouzia Raza^1#^, Franchino Porciuncula^1,2#^, Sangjun Lee^1^, Richard W. Nuckols^1^, Louis N. Awad^2^, Conor J. Walsh^1†^



^1^John A. Paulson School of Engineering and Applied Sciences, Harvard University, Boston, MA 02134, USA.

^2^Sargent College of Health and Rehabilitation Science, Boston University, Boston, MA 02215, USA.

*^#^These authors contributed equally to this work.

†Corresponding author:

Dr. Conor J. Walsh

Paul A. Maeder Professor of Engineering and Applied Sciences at the John A. Paulson School of Engineering and Applied Sciences, Harvard University, Cambridge, MA, USA

[walsh@seas.harvard.edu](mailto:walsh@seas.harvard.edu)

## **Supplemental Material**

**
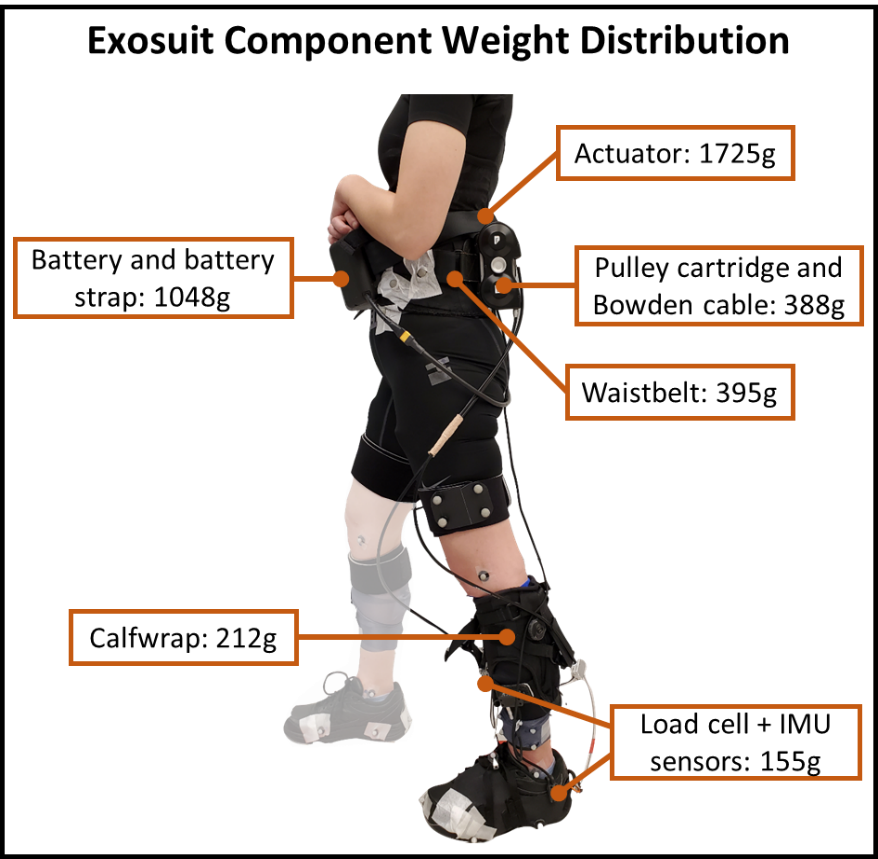

Fig. S1. Exosuit component weights.** Component-wise distribution of mass along the user’s body. Masses of textile components (waistbelt and calfwrap) are provided for the medium sizes, which were used by most participants.

**
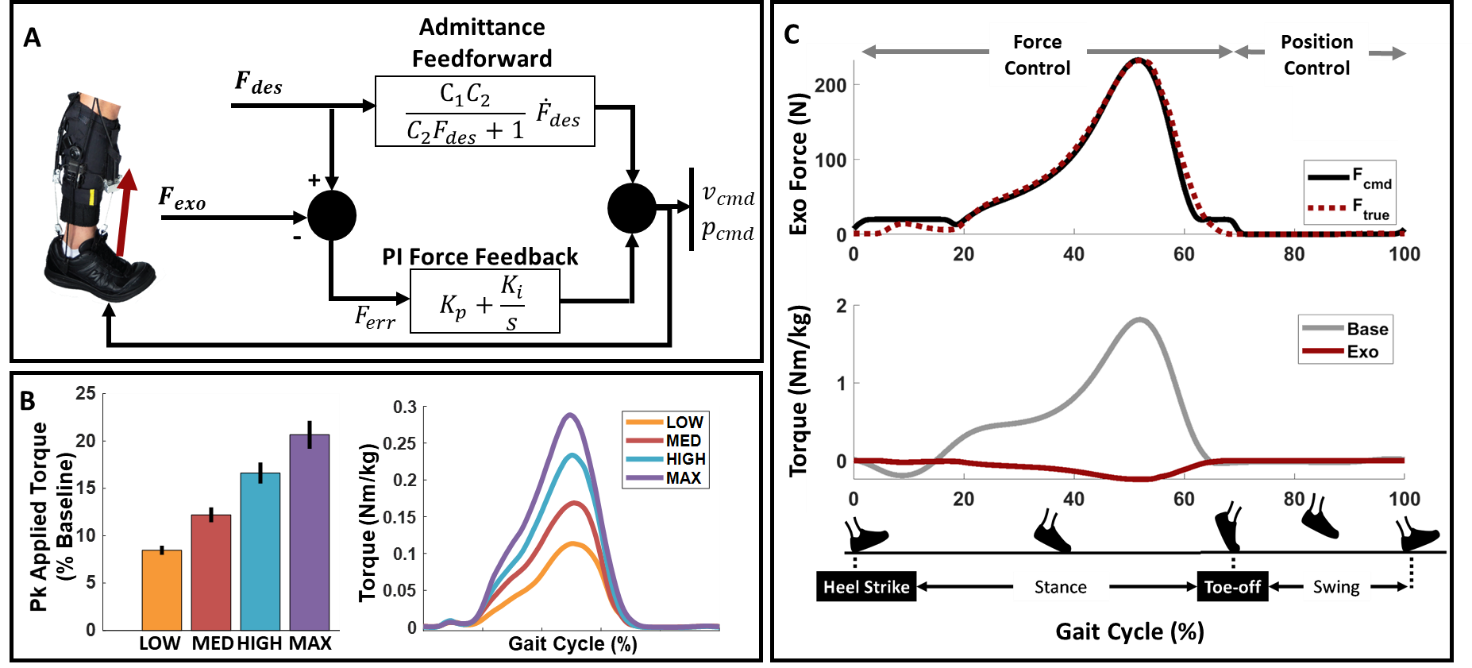
**

**Fig. S2. Force controller architecture and performance. (A)** Controller architecture with PI force feedback and admittance feedforward terms to command motor position and velocity to the actuator. **(B)** Average applied peak exosuit torque across all subjects for each condition (left). Average applied torque profiles across the gait cycle across all subjects for each condition (right). **(C)** Tracking performance for an example stride (top). Resultant exosuit-applied torque and baseline ankle torque for one subject (bottom).

**
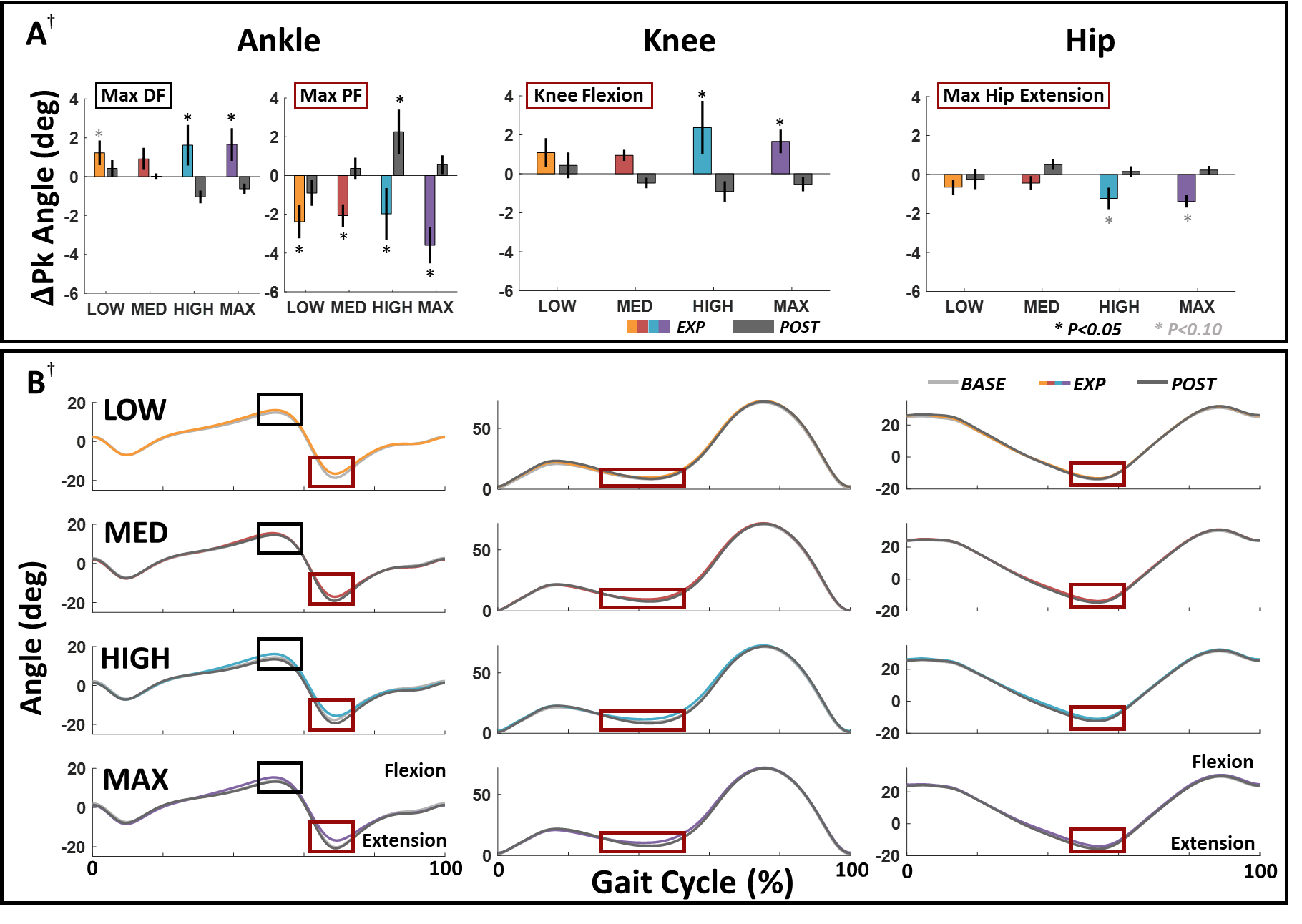
**

**Fig. S3. Joint kinematic response across magnitudes. (A)** Average change in peak dorsiflexion and plantarflexion angle, knee flexion angle during mid-late stance (20-65 %GC), and peak hip extension angle during EXP and POST relative to BASE across all subjects. **(B)** Averaged joint kinematics across all subjects for each condition and section plotted against the gait cycle (0% is heel strike). Local extrema within each boxed region used to generate bar plots in Panel **(A)**. † One subject did not complete the MAX condition (N = 9). All error bars are s.e.m.


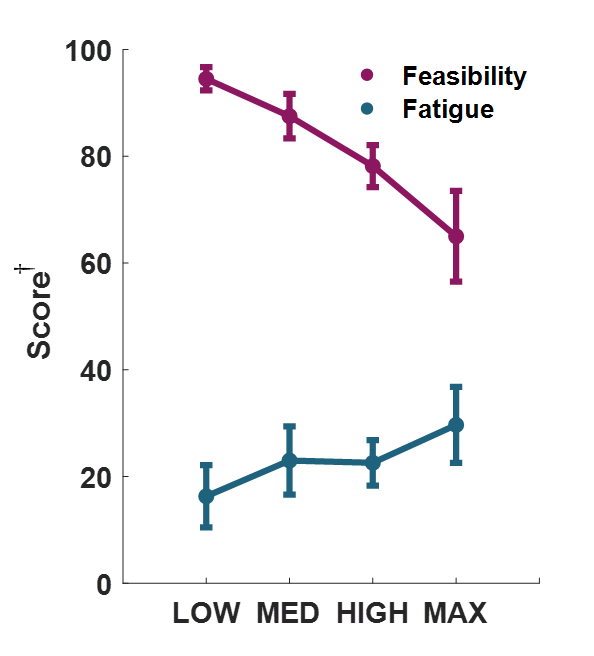


**Fig. S4. Subjective survey data.** Average feasibility and fatigue scores across all subjects in each condition. Feasibility scores indicate the projected ability to walk with the active resistance for 15min continuously, where 0 is impossible and 100 is no foreseeable concern. Fatigue scores indicate the level of fatigue after the active resistance, where 0 is no fatigue. Feasibility relates to comfort while fatigue relates to muscle soreness. †One subject did not complete the MAX condition (N = 9). All error bars are s.e.m.

**Table S1.** Changes in ipsilateral joint kinematics during EXP.

|  | Resistance Condition | | | | | | | |
| --- | --- | --- | --- | --- | --- | --- | --- | --- |
|  | **LOW** | | **MED** | | **HIGH** | | **MAX** | |
| **Variable** | Change | p-value | Change | p-value | Change | p-value | Change | p-value |
| Peak ankle plantarflexion angle (deg) | -2.34 ± 0.86 | **0.012** | -2.07 ± 0.57 | **0.030** | -1.98 ± 1.33 | **0.036** | -3.60 ± 0.93^†^ | **< 0.001** |
| Peak ankle dorsiflexion angle (deg) | 0.91 ± 0.57 | 0.083 | 1.23 ± 0.63 | 0.194 | 1.62 ± 1.04 | **0.023** | 1.65 ± 0.84^†^ | **0.027** |
| Peak knee flexion angle in mid stance (deg) | 1.08 ± 0.75 | 0.176 | 0.94 ± 0.28 | 0.239 | 2.37 ± 1.38 | **0.004** | 1.66 ± 0.61^†^ | **0.049** |
| Peak hip extension angle (deg) | -0.44 ± 0.36 | 0.338 | -0.65 ± 0.38 | 0.519 | -1.23 ± 0.55 | 0.073 | -1.38 ± 0.32^†^ | 0.055 |

Data are mean ± s.e.m. Each value is the difference between EXP and BASE for the corresponding condition and subject. Bolded values indicate significance (p < 0.05). †One subject did not complete the MAX condition (N = 9).

**Table S2.** Effects of exosuit resistance on gait during POST.

|  | Resistance Condition | | | | | | | |
| --- | --- | --- | --- | --- | --- | --- | --- | --- |
|  | **LOW** | | **MED** | | **HIGH** | | **MAX** | |
| **Variable** | Change | p-value | Change | p-value | Change | p-value | Change | p-value |
| Peak ankle torque  (N m kg^-1^) | 0.00 ± 0.01 | 0.967 | -0.02 ± 0.02 | 0.485 | -0.02 ± 0.01 | 0.455 | -0.02 ± 0.01^†^ | 0.552 |
| Peak plantarflexion angle (deg) | -0.9 ± 0.7 | 0.334 | 0.4 ± 0.6 | 0.694 | 2.3 ± 1.1 | **0.017** | 0.6 ± 0.5^†^ | 0.573 |
| Avg soleus stance activation (normalized) | -0.007 ± 0.006 | 0.618 | 0.006 ± 0.007^†^ | 0.648 | 0.013 ± 0.017^†^ | 0.320 | -0.001 ± 0.006^†^ | 0.976 |

Data are mean ± s.e.m. Each value is the difference between POST and BASE for the corresponding condition and subject. Bolded values indicate significance (p < 0.05). †One subject did not complete the MAX condition, and one subject did not have usable EMG data from the MED and HIGH conditions (N = 9).

**Table S3.** Comparison of a passive resistance band and resistive exosuit in a two-subject pilot.

|  | Resistance Condition and Subject | | | | | |
| --- | --- | --- | --- | --- | --- | --- |
|  | **S1** | | | **S2** | | |
| **Variable** | **BAND** | **EXO** | **BAND** | | **EXO** |  |
| Peak bio ankle torque (N m kg^-1^) | 0.230 ± 0.007 | 0.160 ± 0.005 | 0.077 ± 0.014 | | 0.145 ± 0.008 |  |
| Avg bio ankle torque (0-20 %GC) (N m kg^-1^) | 0.032 ± 0.003 | 0.007 ± 0.003 | 0.060 ± 0.005 | | 0.013 ± 0.004 |  |
| Avg bio ankle torque (20-40 %GC) (N m kg^-1^) | -0.088 ± 0.012 | 0.021 ± 0.007 | 0.047 ± 0.016 | | 0.087 ± 0.011 |  |
| Avg bio ankle torque (40-65 %GC) (N m kg^-1^) | 0.073 ± 0.007 | 0.118 ± 0.004 | 0.035 ± 0.011 | | 0.088 ± 0.009 |  |
| Peak hip torque (N m kg^-1^) | 0.278 ± 0.006 | -0.018 ± 0.005 | 0.248 ± 0.015 | | 0.027 ± 0.008 |  |
| Peak torso angle (deg) | 17.4 ± 0.1 | 1.4 ± 0.1 | 14.8 ± 0.2 | | 3.1 ± 0.2 |  |

Data are mean ± s.e.m. Each value is the difference between EXP and BASE for the corresponding condition and subject.

**Table S4.** Effect of instructions on ankle plantarflexor effort in a two-subject pilot.

|  | Instruction Condition and Subject | | | | |
| --- | --- | --- | --- | --- | --- |
|  | **S1** | | | **S2** | |
| **Variable** | **Implicit** | **Explicit** | **Implicit** | | **Explicit** |
| Peak bio ankle torque (N m kg^-1^) | 0.160 ± 0.005 | 0.310 ± 0.007 | 0.145 ± 0.008 | | 0.166 ± 0.009 |
| Avg soleus stance activation (/) | 0.018 ± 0.003 | 0.097 ± 0.004 | 0.033 ± 0.004 | | 0.069 ± 0.004 |
| Avg Ipsilateral VGRF (%BW) | -0.52 ± 0.10 | 1.01 ± 0.12 | -1.90 ± 0.12 | | -0.78 ± 0.18 |
| Avg Contralateral VGRF (%BW) | 0.68 ± 0.10 | -0.73 ± 0.14 | 1.93 ± 0.12 | | -0.89 ± 0.20 |

Data are mean ± s.e.m. Each value is the difference between EXP and BASE for the corresponding condition and subject.
